# On the Action of Ergot of Rye, &c.

**Published:** 1852-07

**Authors:** 


*On the Action of the Ergot of Rye, &c.*—Dr. Arnal, in the *Memoirs of the French Academy of Medicine*, has some interesting observations on this subject, which we find quoted in the Brit. and For. Med. Chir. Review. As the result of extensive trials, he states that the *aqueous extract of the Secale Cornutum* possesses great hemostatic powers in internal hemorrhages. From numerous trials on poultry as well as the human subject, with every variety of dose and preparation, he comes to the following conclusions:

1. The ergot of rye contains a poisonous principle, productive of death, but by no means so energetic as usually represented.—2. Given in the entire grain it acts much less energetically than when powdered.—3. Recent ergot does not act more efficiently than older; but, on the contrary, this last is sometimes the most active of the two.

In order to produce a *summum* of the effect, it is necessary for it to undergo, in the vessels in which it is kept, a peculiar change, which softens it, and imparts to it an odour *sui generis.* Thus it should not be ordered to be powdered just before using.—4. Much greater effect is produced by a certain quantity, in fractional doses, than when given only at twice, probably because less escapes the influence of the digestive organs; one of the effects of divided doses, is to produce a loss of feathers; but in all his numerous experiments, both with large and small doses, Dr. Arnal has never met with anything analogous to the dry gangrene, said to be produced by ergotism in man; but which, seeing that ergot exerts a fluidifying effect upon the blood, he is disposed to attribute to other causes. —5. The ethereal oil of ergot has not proved fatal in his experiments as it did in those of M. Bonjean, and he attributes the issue of these latter to the fluid having entered the air-passages, when it proves rapidly fatal.—6. The watery extiact does not contain poisonous matter, or it does so in such small proportions as to prove injurious only after prolonged use. The toxical principle thus insoluble in ether or water, is found in the residue, which kills animals just as the ergot does.—7. The ergot, however given, is very slow of digestion; aud when given in excess, it produces lesions of the digestive organs. Some of these are found on post-mortem examination to resemble precisely those observed in typhoid fever, and the author exhibits a parallel of the symptoms of typhoid and poisoning by ergot.—8. The ergot modifies the composition of the blood, rendering it more diffluent; and if exhibited long enough in divided doses, it will induce all the symptoms of scorbutus. Nutrition especially suffers from its deleterious action, as is seen by the rapid emaciation that takes place in the animals to which it is given. The aqueous extract exerts a much less modifying power upon the composition of the blood, than do the other preparations.—9. The ergot, in experiments made upon man, reduces the pulse by several beats for some hours; but even by repeated doses, Dr. Arnal has never known these reduced lower than forty-eight, even in the aged.—10. The beneficial effect which ergot exerts upon uterine hemorrhage, has led many to believe that its action is elective, as regards the uterus; but in thirty cases of other internal hemorrhages, in which the *aqueous extract* has been administered by the author, a cure has been effected, or, when the presence of organic disease prevented this, amelioration has been procured. It is, however, not so applicable in all forms of hemorrhage as in uterine. It is rare for active, idiopathic hemorrhage to resist its action for more than twenty-four or forty-eight hours; but when this has become passive, the remedy may even prove mischievous if it be continued too long, or the dose be too large. It is also inefficacious in subjects originally feeble, or exhausted by protracted disease. Even in subjects of good constitution, when given too long in large doses, it may produce bleeding of the gums, and an injurious depression of the circulation. In hemorrhage symptomatic of organic lesion, the ergot acts as a hemostatic, but cannot prevent the return of the bleeding. Yet in the case of hemoptysis, dependent upon tubercle, it may act beneficially, not only by suspending or moderating the *molimen hemorrhagicum*, but also by moderating the inflammatory action of the portion of lung surrounding the tubercular deposit. In the same way, it has proved of constant service in acute bronchitis; and in pneumonia it has rapidly suppressed bloody expectoration, and moderated other symptoms. So well does the author think of it in this point of view, that when the patient's strength requires husbanding, and the pneumonia is not too extensive, he recommends commencing the treatment with the ergot, which, by its deoxidizing agency on the blood and retarding power over the heart's action, is an antiphlogistic, *par excellence;* the debilitating effects which attend other means being not produced by it, or if they should present themselves, ceasing on the discontinuance of the remedy. M. Arnal believes that the experiments of arresting traumatic hemorrhage by the local application of the extract, so favorably reported on by M. Bonjean, require repetition and extension to larger vessels.—11. Ergot in its native state is more active in its operation, but its watery extract is less dangerous.—12. M. Arnal takes the present opportunity of confirming the favorable accounts he formerly gave of the utility of the extract in *chronic engorgements of the uterus.* Some of these cases, however, require a prolonged perseverance in the use of the remedy.

Eighteen cases of hematemesis, epistaxis, &c. &c., are related in illustration. The following is the formula prescribed: Lettuce water, ℥ iv; gum-syrup, ℥j½; aqueous extract of ergot, 15 grains. A tablespoonful every hour and a half.—*Ibid.*

